# Elastic Reconstruction of Chronic Instability of the Distal Tibiofibular Joint in an Obese Patient: A Case Report

**DOI:** 10.7759/cureus.25469

**Published:** 2022-05-30

**Authors:** Meletis Rozis, Christos Vlachos, Elias Vasiliadis, Spyros G Pneumaticos

**Affiliations:** 1 3rd Orthopedic Department, KAT General Hospital, University of Athens, Athens, GRC; 2 3rd Orthopedic Department, KAT Trauma Hospital, University of Athens, Athens, GRC

**Keywords:** ligament reconstruction, tendon graft, chronic ankle instability, syndesmosis, fixation of syndesmosis

## Abstract

An active, obese young patient was admitted to our clinic complaining of chronic ankle pain after fixation of his lateral malleolus fracture. His symptoms consisted of intermittent pain after prolonged walking, swelling, and feeling of instability. His clinical and radiological evaluations indicated chronic mechanical instability of his distal tibiofibular syndesmosis that remained unresponsive to conservative treatment.

Considering his age and activity level, we proceeded to a global syndesmotic reconstruction of the three major syndesmotic ligaments with split-thickness peroneus longus graft. According to this technique, the graft was passed through specific tibiofibular tunnels restoring the native stability and elasticity of the region.

The patient had an optimal postoperative function, with diminished symptoms and increased clinical scores. His late radiological evaluation revealed an anatomic ankle reduction with restoring his normal syndesmotic anatomy compared to his contralateral limb. Regardless of his high BMI, we noticed no further subluxation of his talus, while his general symptomatology was unremarkable at the 12-month follow-up.

In conclusion, elastic reconstruction of the distal tibiofibular joint with split-thickness peroneus longus graft provides excellent results at 12 months regardless of the patient’s BMI. To our knowledge, this is the only technique that restores the three main regional ligaments, simultaneously allowing for close-to-normal biomechanics and providing excellent short-term clinical outcomes.

## Introduction

Instability of the distal tibiofibular joint (DTFJ) has recently gained close attention as it is an easily missed injury that can significantly compromise ankle function. DTFJ can be injured after ankle fractures in up to 15% of the cases with patients suffering from pronation-external rotation (PER) fractures exhibiting a more consistent syndesmotic injury pattern [1,2]. Lauge-Hansen pronation type of injury has been proved to interrupt not only the anterior inferior tibiofibular ligament (AITFL) but also the interosseous (IOL) and posterior tibiofibular (PITFL) ligaments. Those ligamentous injuries have been acknowledged to result in an unstable syndesmosis [3]. Despite varying fixation choices, syndesmotic malreduction has been reported in up to 52% of the surgically treated patients and is considered a substantial predictor of unsatisfactory clinical outcomes after malleolar fractures osteosynthesis [4,5]. Sagi et al. have found that patients with malreduced DTFJ suffered from poor 2-year functional outcomes, and, on the contrary, anatomical reduction of the fibula in the incisura fibularis provides better clinical scores at 12-month follow-up [6,7]. Although patients with malleolar fractures and syndesmotic disruption tend to have worse outcomes than those with an intact DTFJ, syndesmotic fixation has not been proved to alter the clinical course in patients with a supination-external rotation (SER) pattern, compared to PER injuries [8]. Similar reports have also been published by Lehtola et al. where the clinical results in patients with SER fractures were the same at 9.7 years, independently of fixing the syndesmosis or not [9].

Supination fractures tend to interfere less with the syndesmosis, and the prementioned publications report conflicting results on SER fracture patterns. On the contrary, though, pronation injuries cause a major effect on the syndesmotic ligaments resulting in gross instability in either acute or chronic cases [10]. Grass et al. have described their technique for syndesmotic reconstruction in patients with neglected injury and persisting mechanical instability [11]. We present a case of a late DTFJ reconstruction in an obese patient with a PER 4 injury who was not treated for his syndesmosis injury. This report attempts to underline the importance of the syndesmotic fixation in pronation fracture patterns and to evaluate the results of this reconstruction technique in an obese patient.

## Case presentation

A 37-year-old male patient was admitted to our department reporting intermittent swelling of his left ankle along with pain over mild activity and feeling of instability. His clinical history was significant for a lateral malleolus fracture treated with open reduction and internal fixation six months ago, and multiple metatarsal fractures treated conservatively after a motor vehicle accident. His medical history was remarkable with a high BMI of 33 kg/cm^2^. After his initial surgery, the patient was put on a walking boot for six weeks and initiated his rehabilitation with a range of motion (ROM) and strengthening exercises. There were no early complications reported regarding infection or implant failure.

On clinical examination, his ankle and subtalar joint ROM were normal with marked circumferential effusion. A single lateral incision had adequately healed. However, the patient complained of discomfort on his anterolateral ankle, which deteriorated with his foot in dorsiflexion and external rotation. In addition, his fibula was manually unstable in both the sagittal and coronal planes during his clinical examination. As a result, he could not walk and hike for long distances, with the American Orthopedic Foot and Ankle Society (AOFAS) score on admission being 58/100.

**Figure 1:**
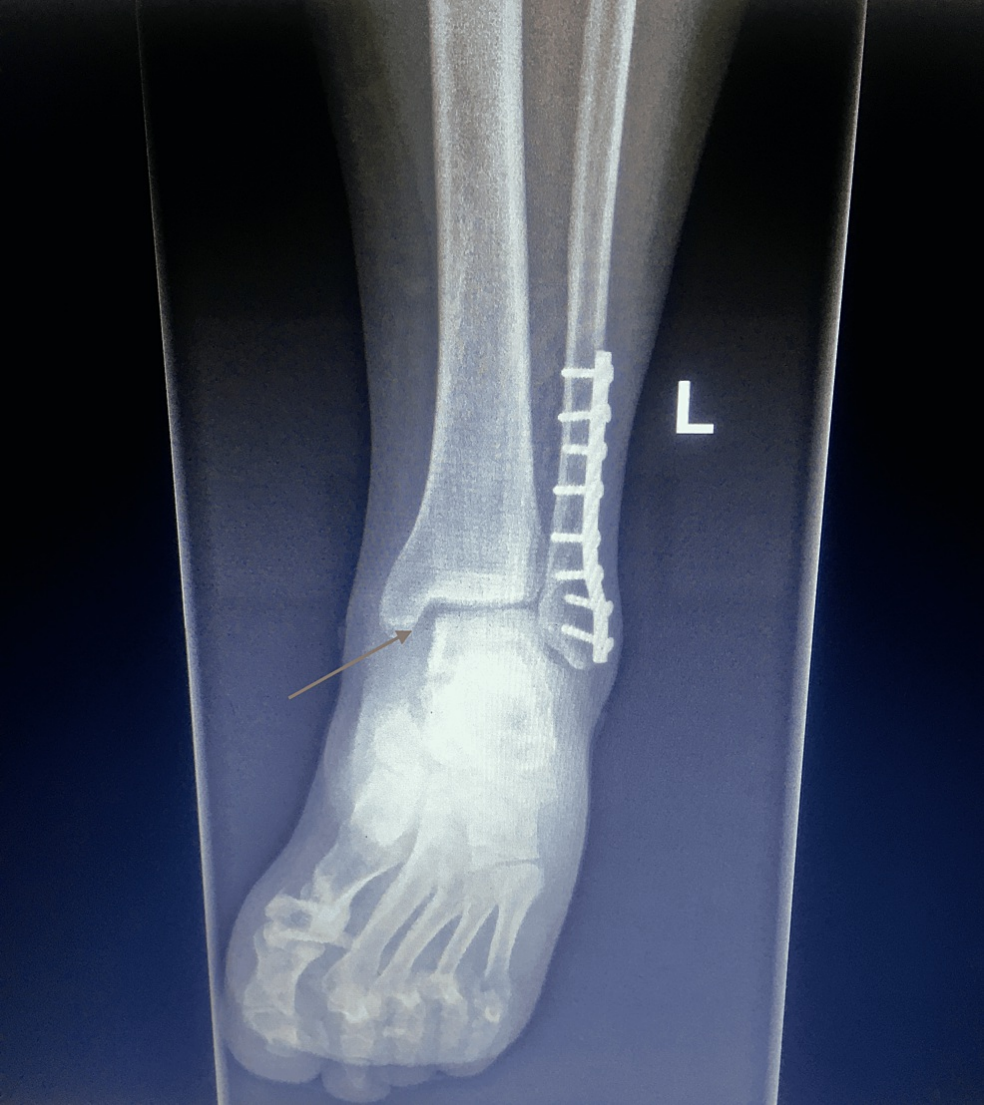
Preoperative roentgenography demonstrates an increased medial clear space and reduced tibiofibular overlap. No syndesmotic screw was used in the initial surgery.

From his clinical examination, a DTFJ instability was presumed. Advocatively, the radiological evaluation revealed a widening of his medial clear space and tibiofibular overlap (Figure 1).

**Figure 2:**
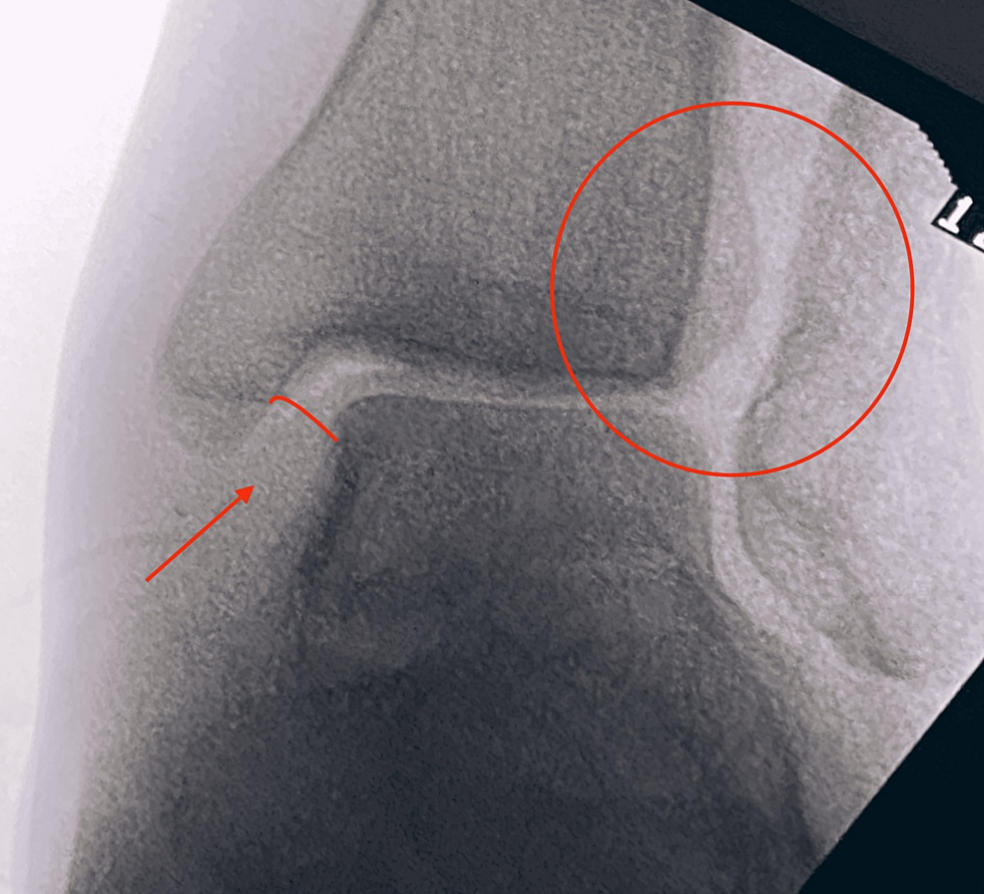
Stress view in dorsiflexion and external rotation. Both medial and tibiofibular clear space has profoundly increased, demonstrating massive instability of the syndesmosis.

After discussing with the patient and having informed consent, we proceeded with a DTFJ triligamentous reconstruction utilizing the technique described by Grass et al. [11]. Under general anesthesia, we removed the plate through the same lateral approach and thoroughly debrided the DTFJ from the anterior and posterior scar tissue. His fractured had properly healed. Next, we performed stress roentgenographies in dorsiflexion and external rotation, demonstrating a gross syndesmotic instability with a further widening of his medial clear space (Figure 2).

We additionally incised the ankle capsule to have direct visualization of the anterior aspect of the incisura fibularis, which was utilized for the intraoperative control of the anatomical reduction of the fibula on the sagittal and coronal plane. The provisional fixation was achieved with a partially threaded tibiofibular screw placed above the reconstruction level. At that stage, intraoperative roentgenographies showed proper talus centralization and a normal medial clear space, so there was no need for a medial gutter debridement.

**Figure 3:**
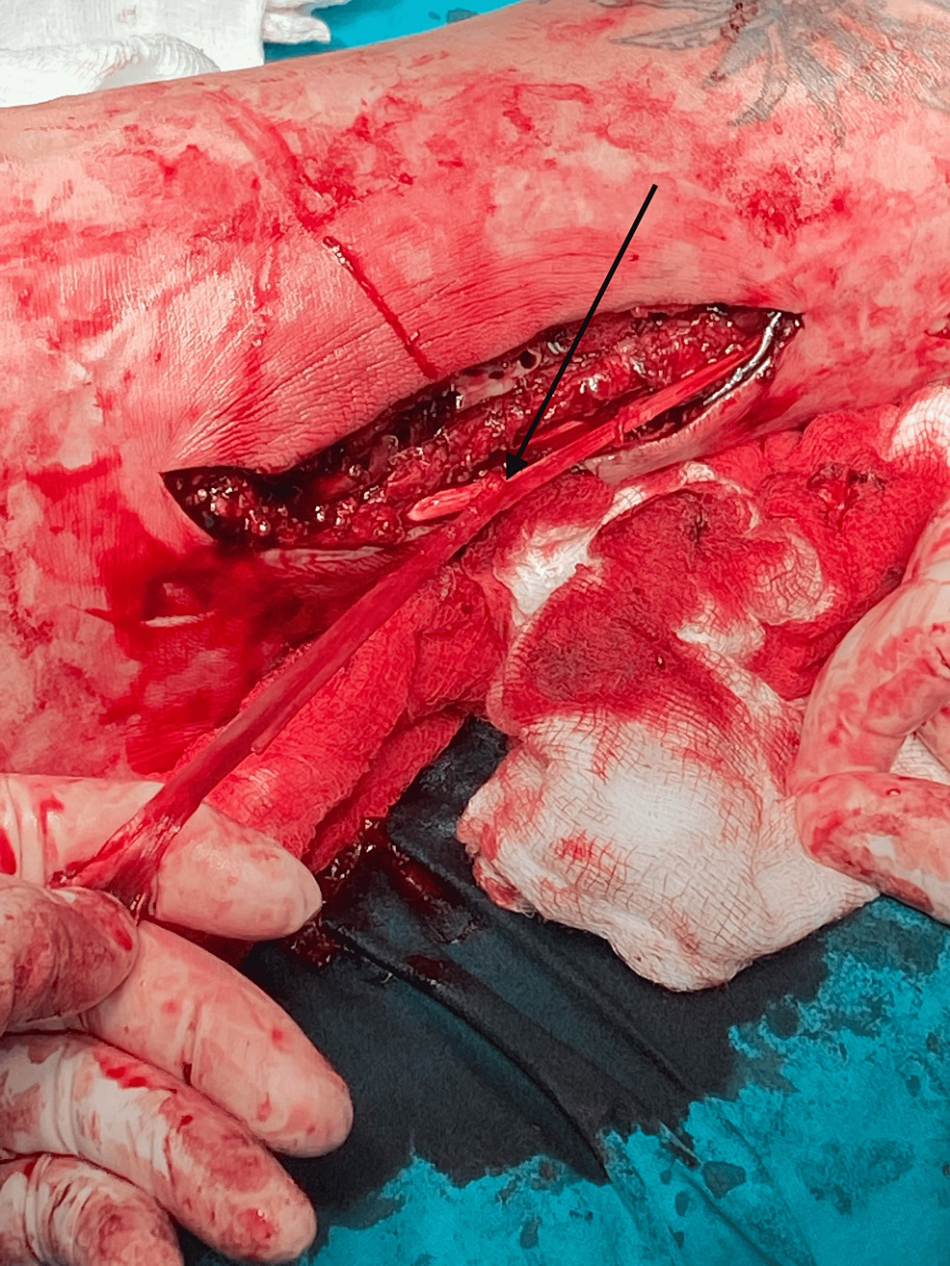
Peroneus longus harvest. A suture is used to split the peroneus longus longitudinally, from the harvest point to 3 cm above the tip of the fibula, gaining a 3.5 mm graft. The harvest point should be as proximal as possible to have an adequate length for the tunnel pass.

From the lateral working approach, we identified the peroneus longus tendon. A small proximal incision was designed at its musculotendinous junction level, and a split-thickness graft was harvested with a nylon suture, maintaining the distal course intact and securing the distal tendinous junction with absorbable sutures (Figure 3).

**Figure 4:**
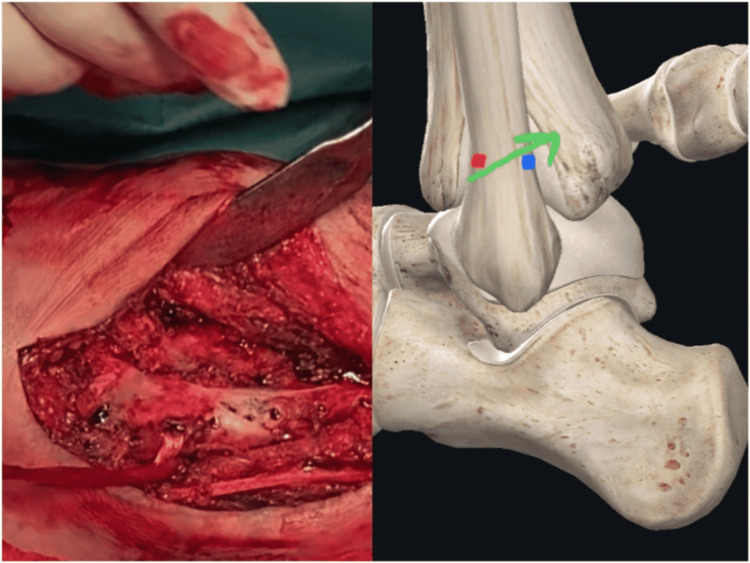
First pass. The graft passes from the first tunnel (green arrow). The entry point (red dot) is at the level of the syndesmosis on the anterolateral aspect of the fibula. The exit point (blue dot) is at the posterior fibular cortex on the same transverse plane as the entry point. After the first pass, the graft is retrieved laterally to be prepared for the second pass.

**Figure 5:**
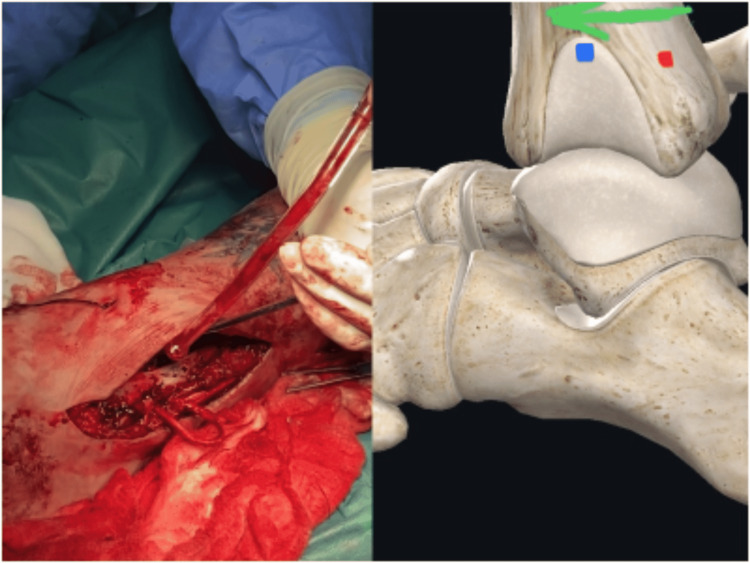
Second pass. At this point, the graft has been retrieved laterally. The graft’s sutures are retrieved through the anteroposterior tunnel (green arrow) with a suture retriever and are pulled out to the anterior aspect of the tibia without pulling the graft. Next, the suture retriever is placed inside the distal tibiofibular tunnel retrieving the sutures from the 2nd-3rd tunnel junction to the tip of the fibula. At this point, the graft sutures pass from the posterior tibia (red dot) exiting to the middle of the incisura fibularis (blue dot).

**Figure 6:**
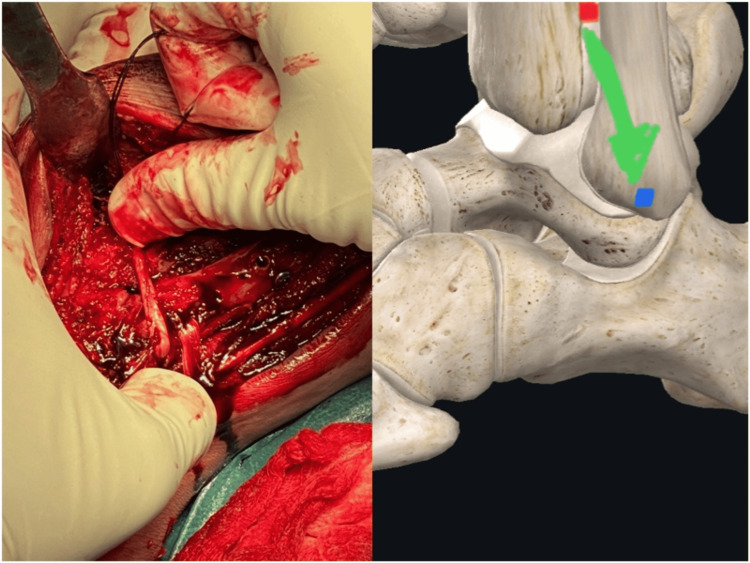
Final pass (green arrow). Pulling the sutures, the graft passes from the posterior tibia to the middle of the incisura fibularis (red dot) and out to the fibular tip (blue dot). Completing the last pass, posterior and interosseous distal tibiofibular ligaments have been restored.

**Figure 7:**
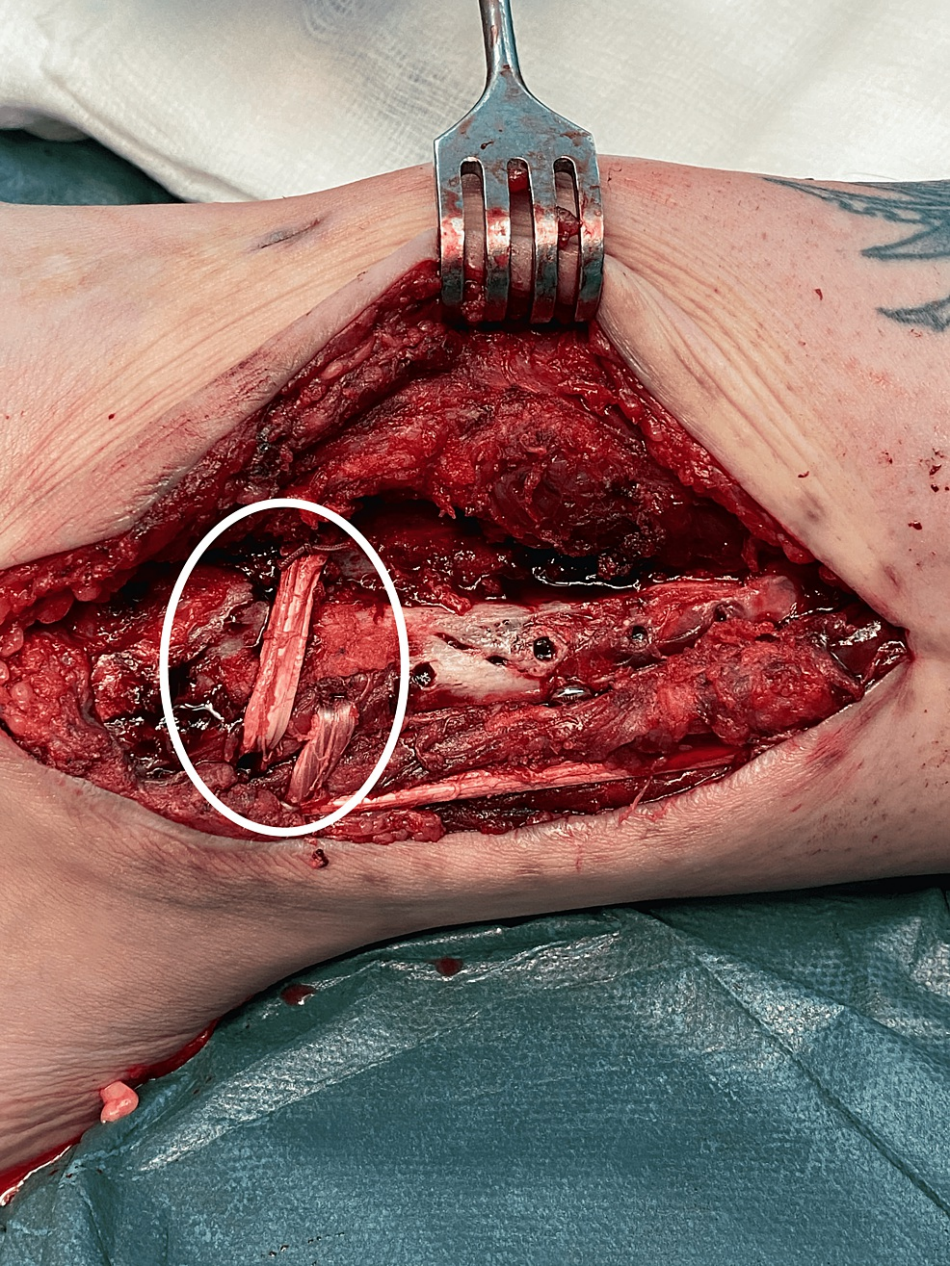
Final fixation to the Tillaux tubercle with an anchor. This step restores the anterior inferior tibiofibular ligament. Final appearance of the reconstruction.

Three 4 mm tunnels were subsequently drilled. The first one started from the lateral fibular aspect resulting posteriorly and inferiorly at the Weber B zone. The second tunnel was drilled anteroposteriorly, perpendicular to the tibia at the same transverse level as the first tunnel. Finally, we created a third tunnel that was approximately 0.5 cm proximal to the fibula tip (anatomical insertion of the AITFL) and aimed toward the second tunnel (tibial tunnel). The tendinous graft was sequentially passed from the first tunnel; it was retrieved from the posterior aspect of the tibia and exited to the fibular tip, restoring the PITFL and IOL in order. The free tendinous edge was finally fixed to the Tillaux’s tubercle with a knotless anchor to restore the AITFL (Figures 4-7).

During the procedure, the graft was manually tensioned after each tunnel pass. A pean mosquito clamp was inserted between the graft and the fibula periosteum to ensure the tightness of the fixation. We always proceeded to the next tunnel only if the clamp could not pass between the graft and the fibula. The surgical wound was closed in a standard fashion, and we put a plaster with the foot in a plantigrade position.

The rehabilitation protocol included a non-weight-bearing plaster for three weeks, followed by a walking boot until the eighth postoperative week. Partial weight-bearing was only allowed after the sixth week. We proceeded with the syndesmotic screw removal in the eighth week and obtained a follow-up both-ankle CT evaluation. The physiotherapy protocol included ROM, strengthening, and proprioception exercises for four weeks. We scheduled his follow-up examinations for the 6th and 12th months.

**Figure 8:**
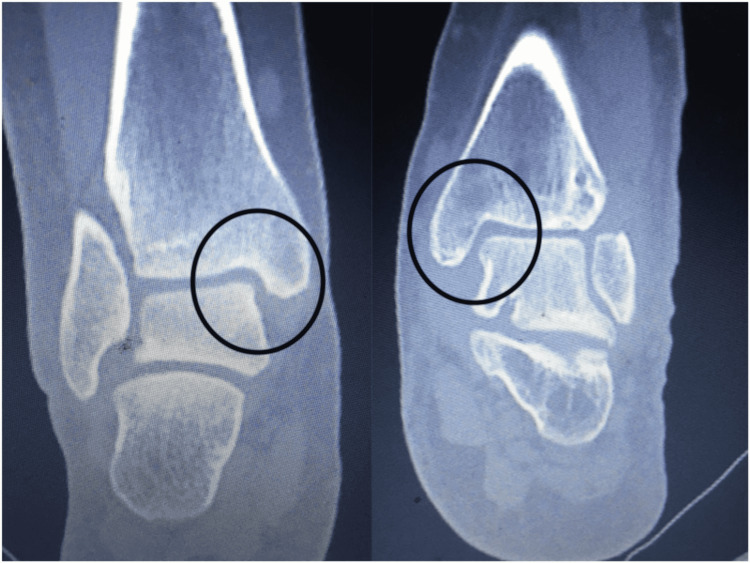
Medial clear space of the right foot was reduced without debridement. The optimal syndesmotic reduction alone restored the normal anatomy.

**Figure 9:**
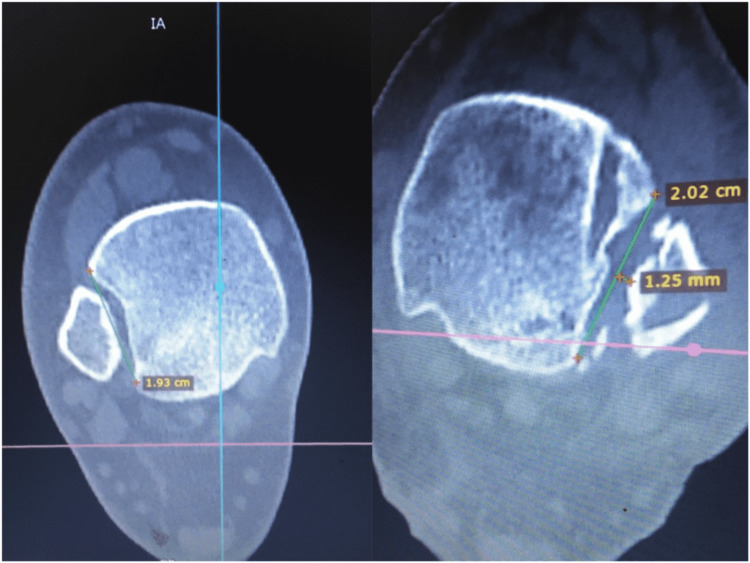
Fibular engagement in the incisura fibularis at the level of 1 cm above the ankle joint. We noticed a 1.25 mm diastasis on the left ankle. The left incisura groove’s depth is bigger and more convex compared to the contralateral side. These changes are advocated for fracture malunion.

**Figure 10:**
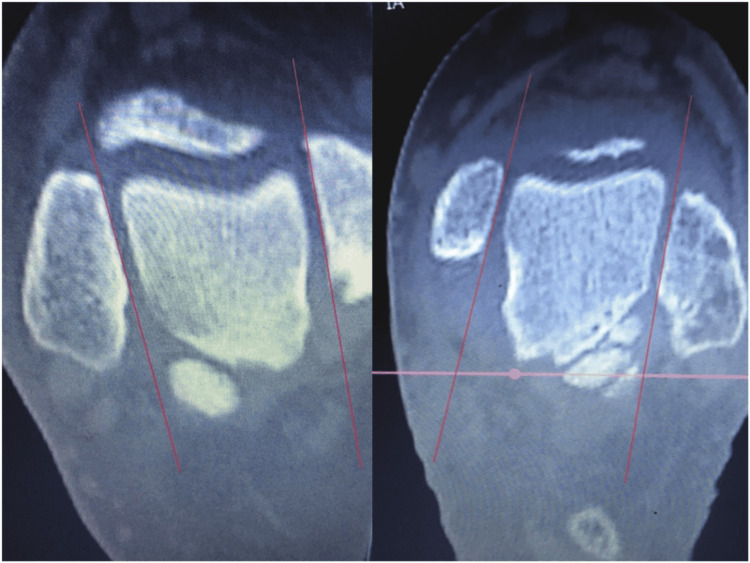
Fibular torsion. The rotation of the fibula was optimal with a deviation of 2 between the two sides.

We report no complications from this procedure. The final CT evaluation revealed an anatomic restoration of the DTFJ, demonstrating optimal values for medial clear space, fibular engagement, and torsion as compared to the contralateral ankle (Figures 8-10).

On a 6-month follow-up visit, the patient claimed to have significantly less pain; he could weight-bear for long distances and had no effusion or joint line tenderness. AOFAS score on the 12th-month visit had increased to 88%. The visual analog scale (VAS) score was at 0/10 in daily activities and up to 2/10 during mountain hiking. In addition, he reported no instability sensation even while slow-running, although he gained more weight during this period, having a current BMI of 33.6. Finally, no arthritic changes were detected at the 12-month follow-up visit in his ankle joint.

## Discussion

We have reported a case of a late DTFJ triligamentous reconstruction in an obese symptomatic patient. We suspected a syndesmotic instability because of the location of his maximum tenderness combined with his joint effusion, but clinical diagnosis can be challenging in general. The examining maneuver of dorsiflexion, external rotation, and compression has been reported to have high sensitivity and excellent intraobserver reliability, especially when eliciting pain throughout the AITFL course [12,13]. In a recent meta-analysis by Netterstrom-Wedin and Bleakley, the specificity and sensitivity of these clinical exams are reported at 97% and 85%, respectively, with the amount of instability correlating to the degree of the ligamentous injury [14,15].

The literature has comprehensively investigated the radiological evaluation of patients suffering from DTFJ instability. From various measuring methods documented, the tibiofibular clear space widening over 5.3 mm has been proved to be a reliable method for the diagnosis [16]. In addition, this measurement seems to be less interfered with by the rotational position of the ankle toward the radiological beam, thus providing reassuring interpretation safety [17]. We had a clear indication of DTFJ widening in our patient, and this was due to the concomitant medial ligamentous injury resulting in talus lateralization. Nevertheless, in cases of intact deltoid, this talar shift may not be evident even with a grossly unstable syndesmosis [18]. In those cases, the conduction of radiological stress views under sedation, with the foot in dorsiflexion and external rotation, can reproduce the instability and validate the diagnosis [19]. In the latest years, the increasing usage of ankle arthroscopy has been a reliable diagnostic tool. More specifically, Lui et al. have reported that performing the external rotation test under direct arthroscopic visualization has greater sensitivity than the radiological external rotation test [20]. Another diagnostic maneuver has been described by Wagener et al. [21]. The authors have found that inserting the arthroscopic probe in the incisura fibularis is an excellent diagnostic indication of syndesmotic disruption.

We confirmed the anatomical restoration of the fibula using two methods. First, we incised the anterolateral capsule intraoperatively to expose the anterior incisura fibularis. This approach helps to reposition the fibula properly as the surgeon can inspect the anterior border and further assess the fibula rotation by opposing the lateral malleolus cartilage to the lateral talar cartilage [22]. Finally, the anatomical restoration was evaluated by a CT scan with our measurements calculated at the level of 1 cm above the tibial plafond as described by Levack et al. [23]. In our experience, exposing the trichondral space (lateral talus, lateral tibial plafond, lateral malleolus) is crucial to enhance the reduction accuracy without needing supplementary intraoperative radiological evaluation.

Although clinical and radiological examinations can provide an assured diagnosis, the extent of the ligamentous injury cannot be set. On the ground of combined DTFJ and medial gutter widening, we decided to reconstruct all three major ligaments of the syndesmosis. Recent research emphasizes the role of the IOL and the AITFL as the primary restrictors [24]. Nevertheless, since obesity is an obvious predisposing factor to early DTFJ ligamentous failure, our patient’s high BMI was regarded as a contraindication for a selective reconstruction [25].

Many surgical options are reported for treating chronic DTFJ instability and are categorized into elastic and stiff techniques. Stiff restoration includes the syndesmotic debridement and screw fixation and the DTFJ fusion, with the last one being a salvage procedure when other options have failed [26,27]. Nevertheless, since the fibula moves relatively to the tibia during normal gait, non-elastic fixation compromises normal biomechanics. Furthermore, studies comparing screw stabilization versus tight-rope have shown that suture buttons tend to have a more elastic in-vivo behavior than screws that approaches the native ROM [28,29]. From the elastic fixation options, anterior tibiofibular ligament (ATFL) reimplantation and the bone block advancement techniques were rejected as they require good AITFL remnants quality and they cannot address the IOL and PITFL [30,31]. Finally, Morris et al. had excellent results in eight patients treated with AITFL and IOL reconstruction using hamstring autograft [32]. Although this can be an optimal and technically less demanding alternative, the impact of obesity has not been studied in the long term.

## Conclusions

The global syndesmotic reconstruction with the split peroneus longus autograft proved excellent results in this high BMI patient suffering from neglected syndesmotic instability. Although demanding, this technique achieves optimal reduction and elastic restriction in all planes, having excellent clinical outcomes regardless of the duration of the symptoms. In addition, it allows a close-to-normal fibula movement during gait phases, compared to several other options described in the literature. Tunnel drilling is of great importance. They should be placed close to the anatomic origins of the three major syndesmotic ligaments and an adequate width will allow for smooth passing without compromising the graft’s edges. The graft’s width of 3.5 mm offered adequate mechanical strength and stability, and the excessive patient’s weight did not deem any clinical problems. Thus, high BMI should not be regarded as a contraindication for late reconstruction with split-thickness peroneus longus graft. Although promising, further research with more patients is needed to evaluate this technique’s safety and efficacy in the obese population. In addition, longer follow-up of these patients is needed to investigate further the overall impact of syndesmotic reconstruction on ankle post-traumatic arthritis.
